# Effect of the heating rate and premelting process on the melting point and volatilization of a fluorine-containing slag

**DOI:** 10.1038/s41598-020-68210-z

**Published:** 2020-07-09

**Authors:** Zhongyu Zhao, Junxue Zhao, Zexin Tan, Boqiao Qu, Liang Lu, Yaru Cui

**Affiliations:** School of Metallurgical Engineering, Xi’an University of Architecture and Technology, Xi’an, 710055 People’s Republic of China

**Keywords:** Thermodynamics, Chemical physics, Marine chemistry

## Abstract

The melting point and volatilization characteristics of a fluorine-containing slag were investigated under different heating rates and premelting processes. The “hemisphere method” was used to detect the melting point, and the results showed that the measurements for the fluorine-free slag increased with increasing heating rate, and the deviation reached 60 °C and was affected by the hysteresis and fractional melting. The measured values and volatilization of the fluorine-containing slag decreased with increasing heating rate. The weight loss reached 16.8%, and the melting point deviation reached 90 °C, which was primarily affected by the volatility. The melting point of the synthetic fluorine-containing slag was 70 °C higher than that of the premelted slag due to flux volatilization of 8.3%. Scanning electron microscopy-energy dispersive spectroscopy revealed a large amount of CaF_2_ on the surface of the melted slag. The internal crystals in the synthetic slag were mainly diamond-shaped calcium fluoroaluminate (3CaO·3Al_2_O_3_·CaF_2_) and those in the premelted slag were needle-shaped cuspidine (3CaO·2SiO_2_·CaF_2_) that formed during secondary crystallization. Two factors that impacted the volatilization were proposed: one is the content of free CaF_2_, and the other is the slag structure. Based on these factors, volatilization models for synthetic and premelted fluorine-containing slags were established. This work is of significance for relevant measurement specifications to ensure the repeatability and comparability of the physicochemical properties, especially for volatile-containing slags.

## Introduction

The properties of metallurgical slag play an important role in determining the smelting temperature, controlling the smelting reaction, metal solidification and inclusion removal. Therefore, it is essential to obtain accurate data about the metallurgical properties of smelting slag, not only to implement metallurgical processes successfully but also to optimize the processes. Many methods for analyzing the performance of metallurgical slag have been widely adopted, such as determining the melting point with the “hemispherical” method and determining the viscosity with the “rotary cylinder method”^1^ (see supplementary Table S1). Many significant results have been achieved by these methods. Arefpour and Anisimov adopted the rotary cylinder method to investigate the effect of CaF_2_ on the viscosity and the structure of mold powder^2,3^. Zhao and Shi used hemispherical method to analyze the melting point, slag formation and crystallization of electroslag^4–6^. Susa and Ju investigated the effects of CaF_2_ and Na_2_O on electrical conductivity by electric bridge method^7,8^. Liu and Cheng analyzed the influence of slag components on the surface tension of the mold powder by suspension link method^9,10^. However, are these methods suitable for all kinds of metallurgical slag? What are the factors that affect the test results and how much is the deviation? All of these issues should be discussed.

It is well known that the melting point is one of the most important parameters for the metallurgical properties of slag, and the measuring process is often accompanied by heating for a long time until the sample decreases to half and appears hemispherical, which is called the “hemisphere method”. However, if the slag contains volatiles, such as fluorides, zinc oxides, and lead oxides, component volatilization is inevitable at high temperatures, and that will lead to changes in the composition^11–16^. This paper focuses on the influence of the heating rate and premelting process on the melting point and volatilization of fluorine-containing slag with a measured performance and an established model.

## The effect of heating rate on the melting point and volatilization

The fluorine slag samples were prepared with pure reagents according to the composition from steel plants in Table 1. To compare the effect of the heating rate on the melting point and volatilization, the fluorine-free slag samples were prepared with pure reagents in the low-melting-point zone of the CaO-SiO_2_-Al_2_O_3_ phase diagram (see supplementary Figure S1), as shown in Table 1.

**Table 1:** Slag composition.

Composition	Al_2_O_3_	MgO	SiO_2_	CaF_2_	CaO
Fluorine-containing slag (%)	29.33	2.57	9.15	30.12	28.83
Fluoride-free slag (%)	18	–	42	–	40

**Figure 1:**
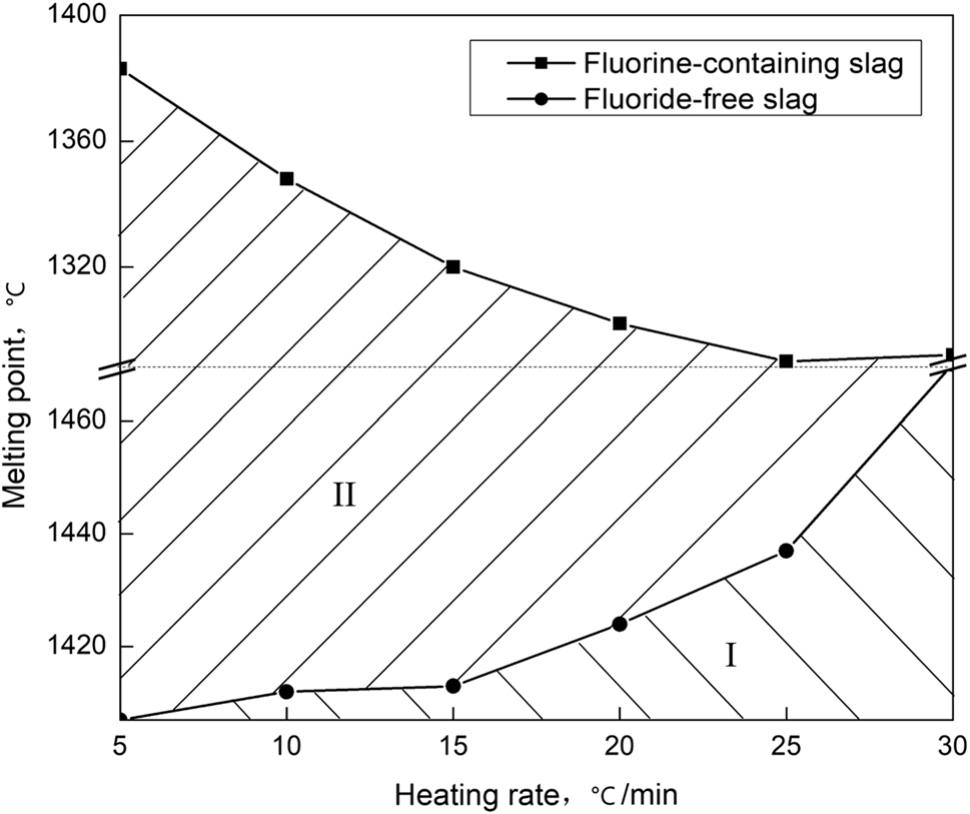
Effect of different heating rates on the melting point of fluorine-containing slag and fluorine-free slag.

A CQKJ-II slag melting temperature characteristic tester was used to detect the melting point by the “hemisphere method”, with Ar gas protection at a flow rate of 50 ml/min. Moreover, the samples before and after the test were weighed. The results are shown in Table 2 and Fig. 1.

**Table 2:** Melting point detection.

Slag	Heating rate (°C/min)	5	10	15	20	25	30
Fluorine-containing slag	Test 1 (°C)	1,380	1,347	1,317	1,308	1,295	1,297
	Test 2 (°C)	1,385	1,348	1,322	1,298	1,286	1,287
	Weight loss (%)	16.8	8.3	7.5	6.2	5	4.9
Fluoride-free slag	Test 1 (°C)	1,407	1,412	1,413	1,417	1,430	1,476
	Test 2 (°C)	1,406	1,415	1,416	1,422	1,437	1,468
	Weight loss (%)	1.6	1.7	1.6	1.5	1.4	1.5

Comparing the melting points shown in Table 2, it can be seen that the measured values were reliable within the deviation for two repeated tests of less than 5 °C. The weight loss data during melting point detection showed that the fluorine-containing slag had a significant weight loss that reached 16.8%.

First, it was observed that the melting point of the fluorine-free slag increased with increasing heating rate, which can be analyzed from two aspects. On the one hand, it was affected by the detection method. If the heating rate was too high, the measured value would exceed the hemispherical temperature, resulting in an elevated melting point. This phenomenon was generally called the “hysteresis” of the melting point detection, and the larger the heating rate was, the more obvious this hysteresis performance. On the other hand, combined with the theory of slag fractional melting characteristics, the low melting point components of the synthetic slag melted preferentially, and the high melting point components followed, resulting in an overall melting temperature that was higher than the slag melting point. The higher the heating rate was, the more insufficient the slag forming process, and the more significant the fractional melting characteristic, as zone I shows in Fig. 1. The melting point deviation for the fluorine-free slag was approximately 5 °C at a heating rate of 10–15 °C/min, which can be up to 60 °C at 30 °C/min. Therefore, the heating rate was usually set to 10–15 °C/min during melting point detection.

Then, the fluorine-containing slag exhibited an opposite trend to that of the fluorine-free slag for the melting point measurement. The melting point increased as the heating rate increased, and this was inexplicable according to the above theory of "fractional melting" or "hysteresis". However, there was significant volatilization during the melting point detection process for the fluorine-containing slag, and the slower the heating rate was, the longer the heating time, and the greater the weight loss. Related research has shown that the volatile species are mainly fluorides^14–17^. If the volatile was regarded as CaF_2_, the loss of CaF_2_ in the slag could exceed 50% at most. The greater the fluoride loss was, the worse the flux (CaF_2_) effect, and the higher the measured value, resulting in the deviation of the measured value reaching 90 °C. Taking the melting point curve of fluorine-free slag as a baseline, if the slag was not affected by the volatility, the two curves should coincide or have similar trends. Therefore, the difference between the two curves (zone II) can be regarded as the volatile influence zone. It can be seen that the smaller the heating rate was, the longer the heating time, the larger the intermediate difference, and the greater the volatility effect.

In summary, the melting point of the fluorine-containing slag was affected by the hysteresis, fractional melting and volatility at different heating rates, and the deviation can be regarded as the superposition of zone I and zone II. It can be seen in Fig. 1 that the deviation can reach 150 °C at a heating rate of 5 °C/min. Moreover, the same trend was obtained during the melting point detection for the fluorine-containing mold flux at different heating rates (see supplementary Figure S2).

## The influence of the premelting process on the melting point and volatilization

### The effect of the premelting process on the melting point

Metallurgical slag can be divided into synthetic slag and premelted slag according to different preparation techniques. The premelting process involves heating or premelting the slag components so that the slag can melt rapidly, the constituents can be relatively uniform, and fractional melting can be effectively avoided. In theory, the premelting process only changes the phases and melting speed of slag but not the melting point. However, if the premelting technique is suitable for fluorine-containing slags and whether it affects the melting point need to be determined.

**Figure 2:**
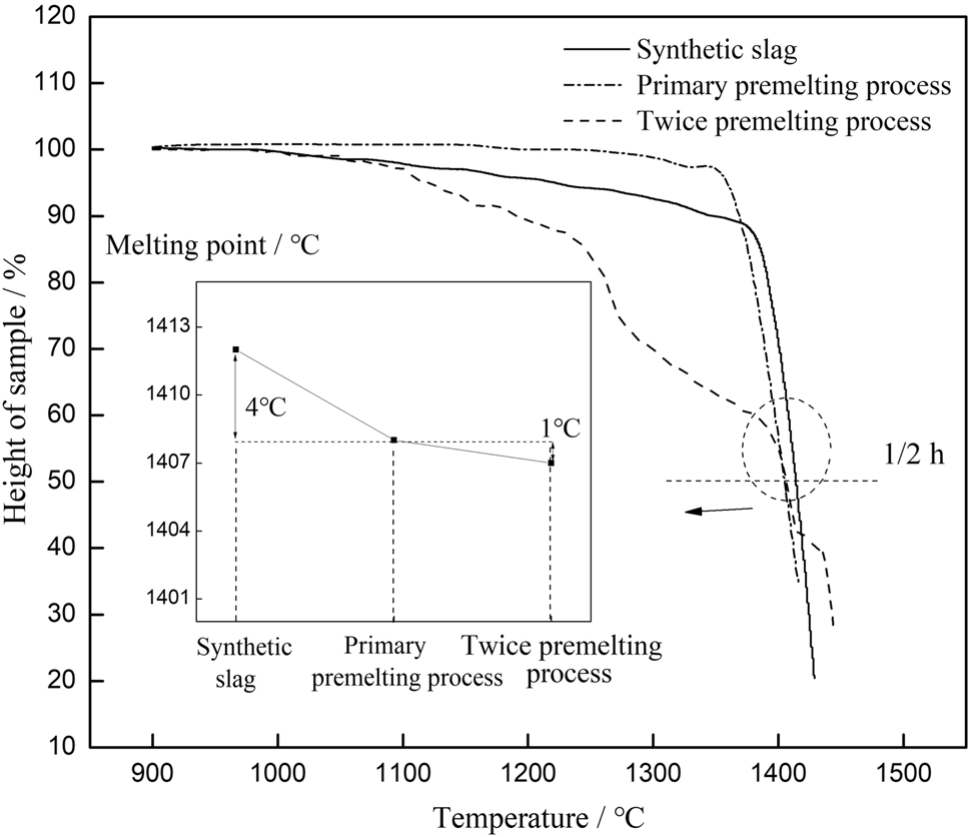
Synthetic and premelted fluorine-free slag melting point test.

**Figure 3:**
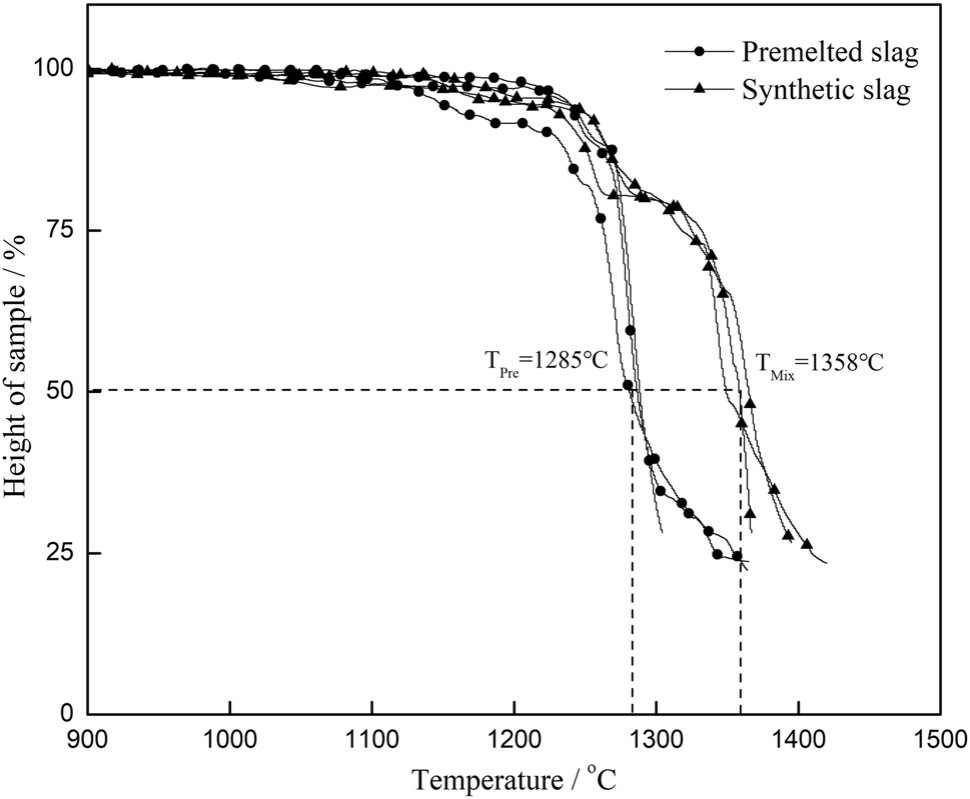
Melting point test for synthetic and premelted fluorine-containing slags (*Note*: the synthetic slag was prepared with chemical reagents according to XRF analysis of premelted fluorine-containing slag to ensure a consistent composition).

The melting point changes before and after the premelting process for the above fluorine-containing slag and fluorine-free slag was determined with the same equipment at a heating rate of 10 °C/min. The results are shown in Figs. 2 and 3.

Figure 2 shows that the difference in the melting points was within 5 °C for the synthetic and premelted fluorine-free slag, and the influence of the premelting treatment on the melting point was not significant. For the fluorine-containing slag, the melting point deviation between the synthetic and premelted slag of the same composition was 70 °C, as shown in Fig. 3. Therefore, the main factor that changed the melting point of the fluorine-containing slag cannot be the premelting process.

### The volatilization characteristics of the synthetic and premelted fluorine-containing slags

**Figure 4:**
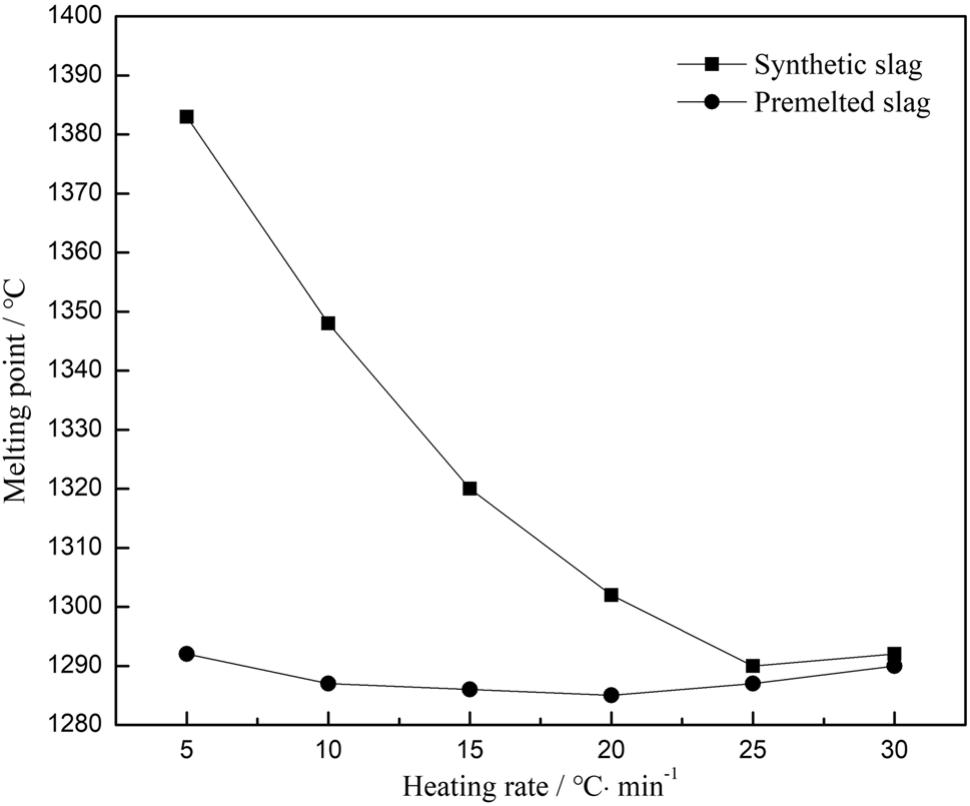
Effect of different heating rates on the melting point of the fluorine-containing slag.

The melting points of the synthetic and premelted fluorine-containing slags above were measured at different heating rates, and the results are shown in Fig. 4.

The melting point of synthetic slag was obviously higher than that of the premelted slag, and the deviation increased as the heating rate decreased, which indicated that the influence of fractional melting and volatilization on the premelted slag was relatively small compared to that for the synthetic slag. Comparing the trend of two curves, it can be judged that the melting points of both slags were affected by volatility.

**Figure 5:**
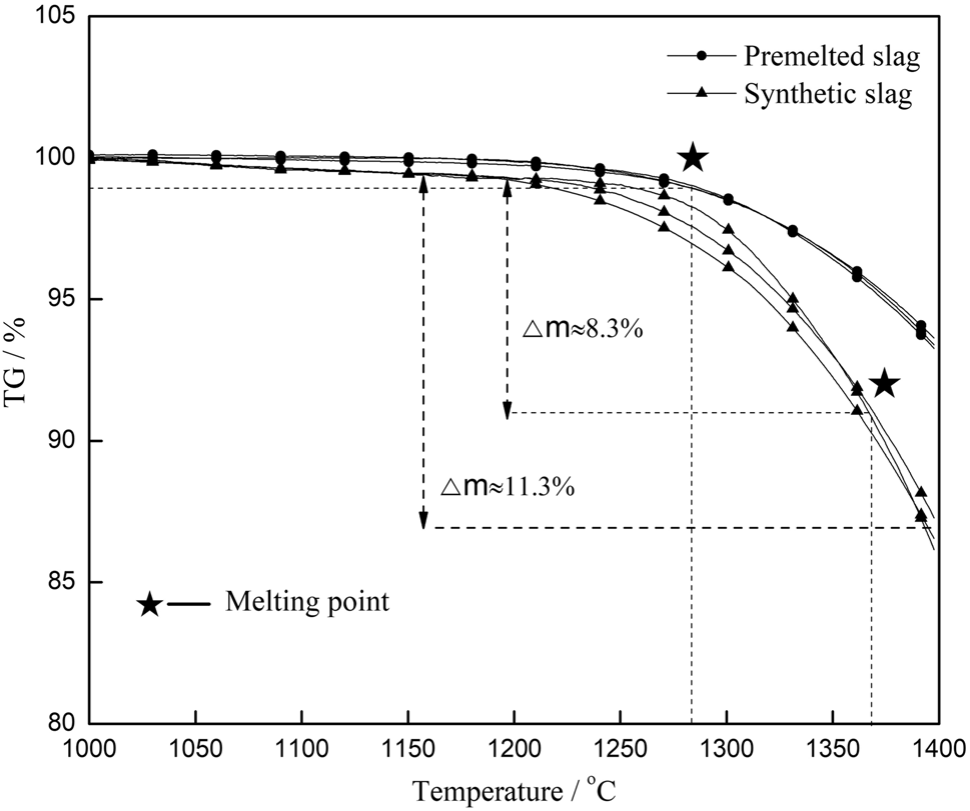
TG test results for the fluorine-containing slag.

An HCT-III thermal analyzer was used to perform the thermogravimetric (TG) analysis of the synthetic and premelted slags with a Pt–Rh crucible. The heating rate was 10 °C/min with Ar gas protection at a flow of 50 ml/min. The results are shown in Fig. 5.

Figure 5 shows that various weight loss values for the fluorine-containing slag occurred during the melting point detection test. The weight loss of synthetic fluorine-containing slag was 8.3% at the melting point, while the volatilization of the premelted slag was almost negligible. Therefore, the melting point of the synthetic fluorine-containing slag was increased due to the increased weight loss of flux, and the deviation increased with a decrease in the heating rate.

### The crystals and phases in the synthetic and premelted fluorine-containing slags after melting

**Figure 6:**
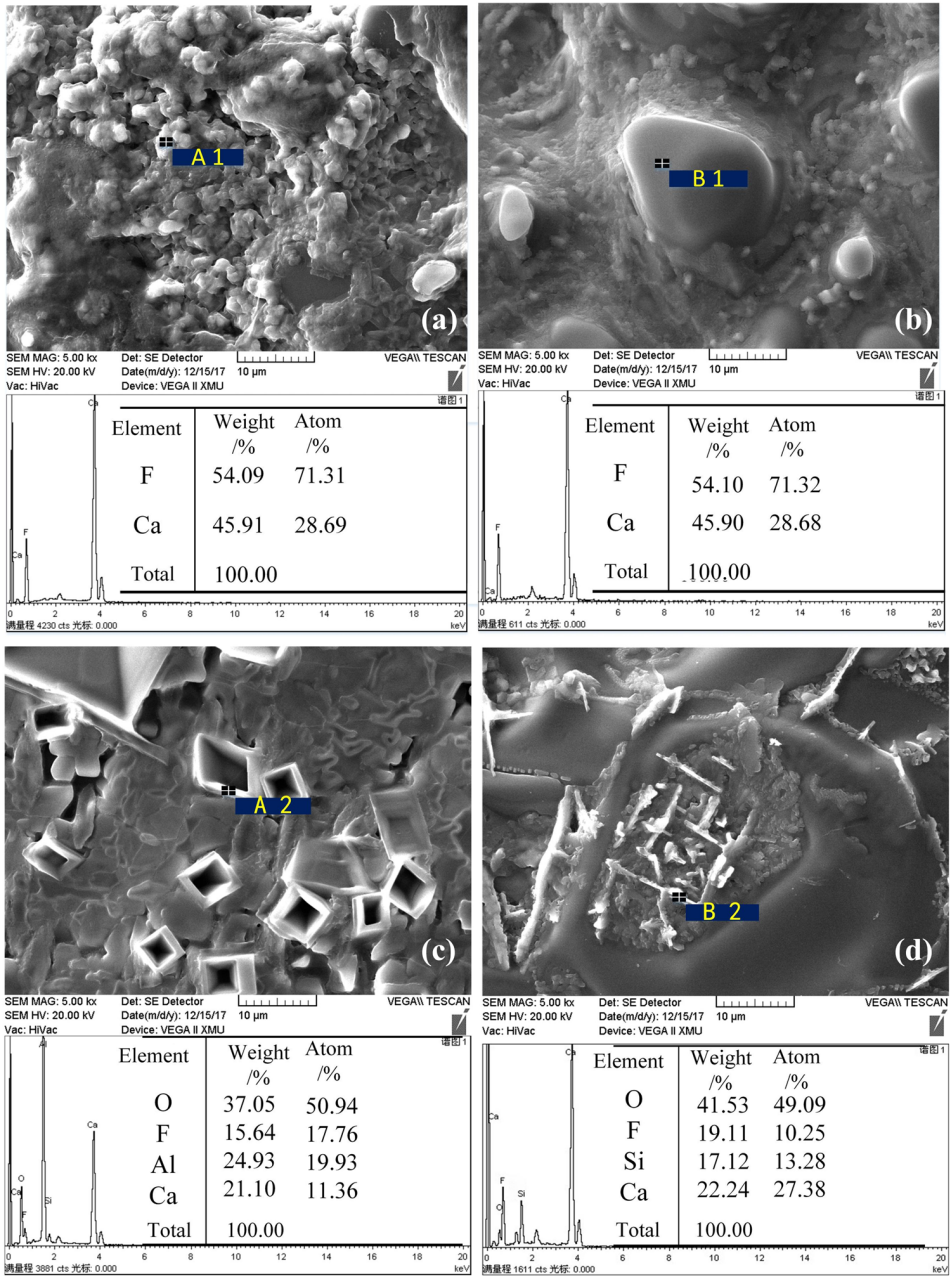
SEM–EDS analysis of melted fluorine-containing slag: (**a**) surface of the synthetic slag, (**b**) surface of the premelted slag, (**c**) interior of the synthetic slag, and (**d**) interior of the premelted slag.

To explore the slag melting and forming processes of the synthetic and premelted fluorine-containing slags, a tube furnace (see supplementary Figure S3.) was used for a roasting test, and the experimental conditions were completely in accordance with the melting point detection above. The phase observation and SEM–EDS analysis of the melted samples were carried out, as shown in Fig. 6.

It can be seen from the SEM–EDS analysis above that the main component of the slag surface was CaF_2_, and the surface of the synthetic slag was relatively loose compared to that of the premelted slag. The difference between them is obvious when comparing the structure and composition of the slag interior. The crystals in the interior of synthetic slag were mainly diamond-shaped calcium fluoroaluminate (3CaO·3Al_2_O_3_·CaF_2_). For the premelted slag, the crystals were needle-shaped cuspidine (3CaO·2SiO_2_·CaF_2_) formed by the secondary crystallization of the slag. Therefore, although the initial components of the two slags were consistent, the structures and phases in the melted fluorine-containing slags were quite different, resulting in the different volatile characteristics and composition changes between the synthetic slag and premelted slag.

### Fluoride volatilization model

**Figure 7:**
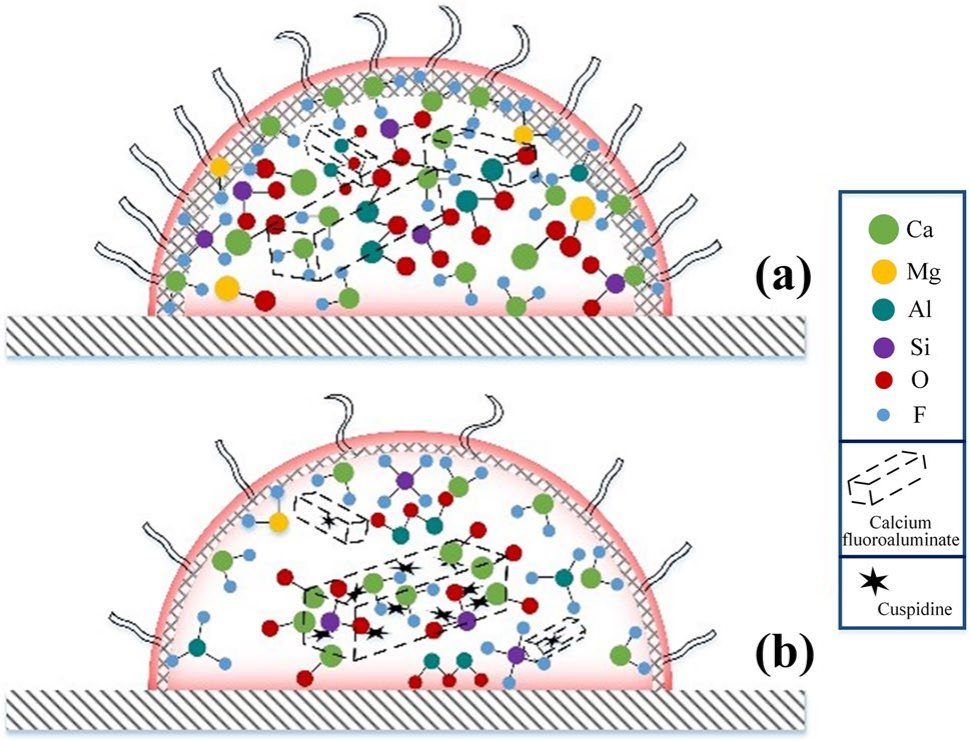
Volatilization model of fluorine-containing slag (**a**) Synthetic slag; (**b**) premelted slag.

In view of the above results, volatilization was the essential factor affecting the melting point of the fluorine-containing slag. Therefore, two factors that impact the volatilization process are proposed. The first is the content of free CaF_2_ in the slag system, which can substantially promote the volatilization of a fluorine-containing slag. There was a large amount of free CaF_2_ in the synthetic slag prepared with chemical reagents, leading to significant volatilization, composition variation, and measured deviation of the melting point. While there was a small amount of free CaF_2_ in premelted slag, most of the CaF_2_ was in the form of calcium fluoroaluminate or cuspidine. The second is the slag structure. The synthetic fluorine-containing slag was obviously volatilized because of the loose surface structure and that of the premelted slag was so dense that the fluoride did not easily escape from the slag surface. Furthermore, the volatilization of the synthetic and premelted fluorine-containing slags can be modeled based on macrostructure and volatile detection (see supplementary Figures S4 and S5), as shown in Fig. 7.

This model showed that for synthetic fluorine-containing slag, the surface structure was loose and full of free CaF_2_, causing volatilization, and the volatiles were CaF_2_ and a small amount of MgF_2_, SiF_4_, and AlF_3_ with internally formed calcium fluoroaluminate. For the premelted slag, the surface structure was dense with less free CaF_2_ and had a weak volatility. Simultaneously, cuspidine was formed through secondary crystallization.

## Conclusions and prospects

1. The melting point of the fluorine-free slag increased with increasing heating rate, and the deviation reached 60 °C due to hysteresis and fractional melting. The melting point and weight loss of the fluorine-containing slag decreased with increasing heating rate. The weight loss reached 16.8%, and the melting point deviation reached 90 °C, which was primarily affected by volatility.
2. The melting point of the synthetic fluorine-containing slag was 70 °C higher than that of the premelted slag due to a flux volatilization of 8.3% when it was heated at 10 °C/min until melting. After contrast and analysis of crystals and phases of two melted slags, there was a large amount of CaF_2_ on the slag surface, and the internal crystal of synthetic slag was mainly diamond-shaped calcium fluoroaluminate (3CaO·3Al_2_O_3_·CaF_2_). For the premelted slag, the internal crystal was needle-shaped cuspidine (3CaO·2SiO_2_·CaF_2_) and formed during secondary crystallization.
3. The volatilization was the essential factor affecting the melting point of the fluorine-containing slag. Two impact factors for volatilization were proposed: one is the content of free CaF_2_ in the slag system, and the other is the slag structure, based on which the volatilization models for synthetic and premelted fluorine-containing slags were established.
4. It is necessary to establish relevant measurement specifications for volatile-containing slags to ensure the repeatability and comparability of the physicochemical properties, such as melting point, viscosity, and conductivity. Moreover, new assays should be developed to eliminate the effects of volatilization on the measured values.

## Supplementary information

Supplementary file1 (PDF 568 kb)

## Data Availability

All data involved in this article are available.

## References

[1] Li ZB (2010). Electroslag Metallurgy Theory and Practice.

[2] Arefpour AR, Monshi A, Saidi A (2013). Effect of CaF2, and MnO on mold powder viscosity and solidification during high-speed continuous casting. J. Refract. Ind. Ceram..

[3] Anisimov KN, Longinov AM, Toptygin AM (2016). Investigation of the mold powder film structure and its influence on the developed surface in continuous casting. J. Steel Transl..

[4] Zhao JX, Ge BL, Cui YR (2016). High temperature properties measurement of slag with higher volatile content. J. Ind. Heat..

[5] Zhao JX, Zhao ZY, Cui YR (2018). New slag for nickel matte smelting process and subsequent Fe extraction. J. Metall. Mater. Trans. B..

[6] Shi CB, Cho J, Zheng DL (2016). Fluoride evaporation and crystallization behavior of CaF–-CaO–Al2O3–(TiO2) slag for electroslag remelting of Ti-containing steels. J. Int. J. Miner. Metall. Mater..

[7] Susa M, Sakamaki T, Kojima R (2013). Chemical states of fluorine in CaF2–CaO–SiO2 and NaF–Na2O–SiO2 glassy slags from the perspective of electronic polarisability. J. Ironmak. Steelmak..

[8] Ju JT, Lv ZL, Jiao ZY (2012). Experimental study on the electrical conductivity of CaF2–SiO2–Al2O3–CaO–MgO slag system. J. Iron Steel Res..

[9] Liu LG, Zhu LG, Wang XJ (2018). Study on relationship between surface tension and viscosity for mold powder. J North China Univ. Sci. Technol. (Nat. Sci. Ed.).

[10] Cheng YH, Wang Y, Li DK (2008). Effect of chemical components of mold flux on surface tension of molten slag. J. Contin. Casting..

[11] Mao HX, Hu HT, Ma GJ (1999). Contamination of fluorine in CC mould powder to environment and countermeasures. J. Steeingmak..

[12] Mills KC (1988). Physical properties of casting powders. J. Ironmak. Steeling..

[13] Chen LY, Wen GH, Yang CL (2015). Development of low-fluoride and titanium-bearing mould fluxes for medium carbon peritectic steel slab casting. J. Ironmak. Steelmak..

[14] Zhao, J.X. Shang, N., Zhao, Z.Y., *et al*. Influence of slag preparation mode on melting point of ESR slag with medium fluoride content. *J. Steeingmak*. **53**(8), 44–48 (2018).

[15] Park JY, Ryu JW, Sohn II (2014). In-situ crystallization of highly volatile commercial mold flux using an isolated observation system in the confocal laser scanning microscope. J. Metall. Mater. Trans. B.

[16] Guo J, Peng K, Yi L (2014). Study on properties of Al2O3-CaO–SiO2–CaF2–MgO Slag system. J. Appl. Mech. Mater..

[17] Li J, Zhang L, Tan Y (2014). Research of boron removal from polysilicon using CaO–Al2O3–SiO2–CaF2 slags. Vacuum.

