# Supplementary material for: Effect of the heating rate and premelting process on the melting point and volatilization of a fluorine-containing slag
Source: Sci Rep. 2020 Jul 9;10:11254. doi: 10.1038/s41598-020-68210-z (PMC7347843; doi:10.1038/s41598-020-68210-z)
Supplement: Supplementary file 1 — Supplementary file1 (PDF 568 kb) [file 41598_2020_68210_MOESM1_ESM.pdf]

Supplementary for Effect of the Heating Rate and Premelting Process on the Melting Point and Volatilization of a Fluorine-containing Slag

ZHAO Zhongyu ZHAO Junxue TAN Zexin QU Boqiao LU Liang CUI Yaru*

School of Metallurgical Engineering, Xi'an University of Architecture and Technology, Xi'an 710055, P.R. China

Figures

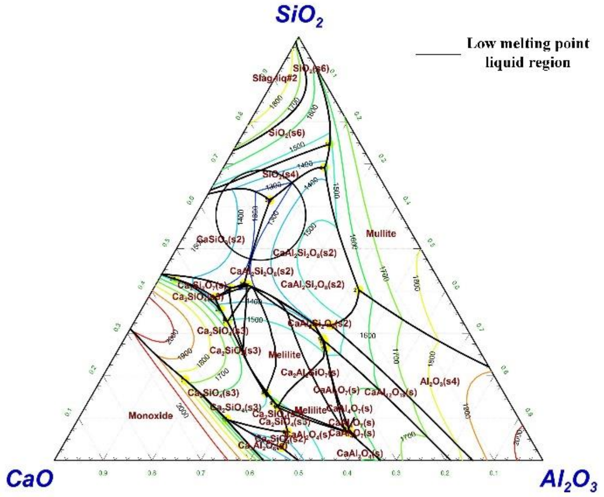

Figure S1 $CaO-Al_2O_3-SiO_2$ phase diagram

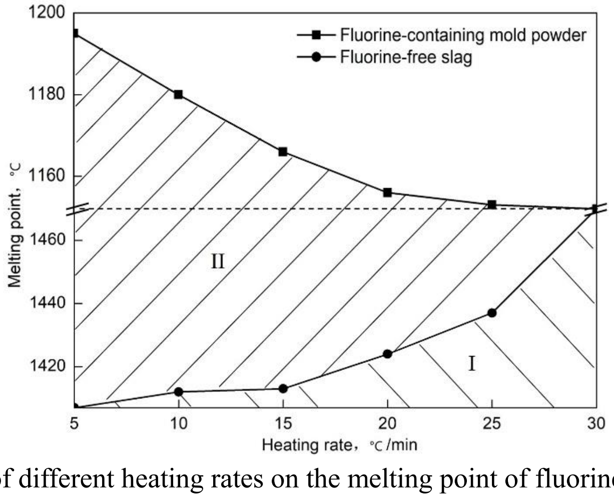

Figure S2 Effect of different heating rates on the melting point of fluorine-containing mold powder and fluorine-free slag

* Corresponding author: E-mail: Zhaojunxue1962@126.com

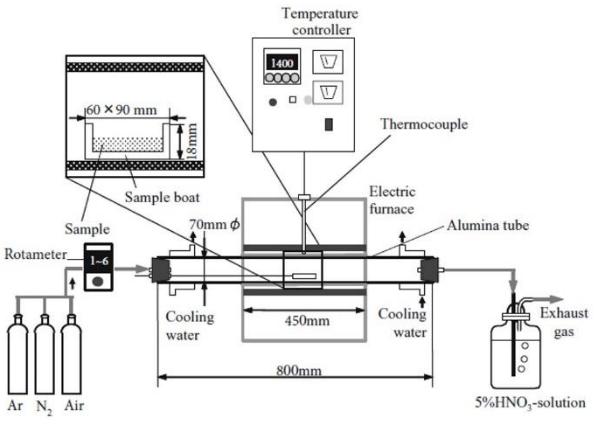

Figure S3 Tube furnace for Roasting test

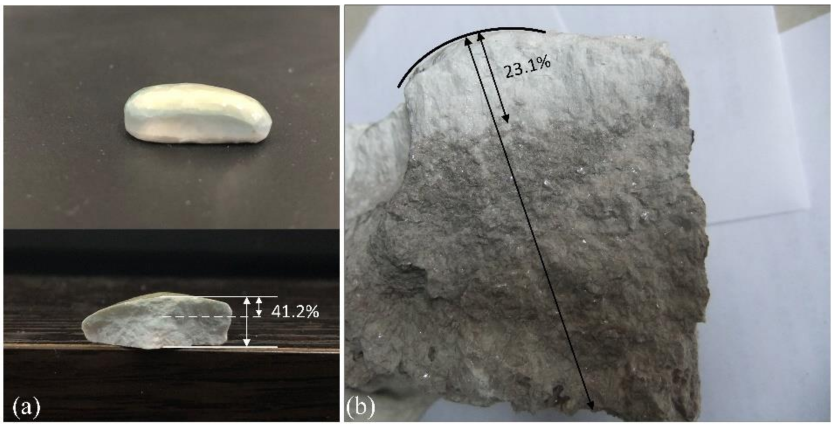

Figure S4 Slags after roasting

(a) synthetic slag; (b) pre-melted slag

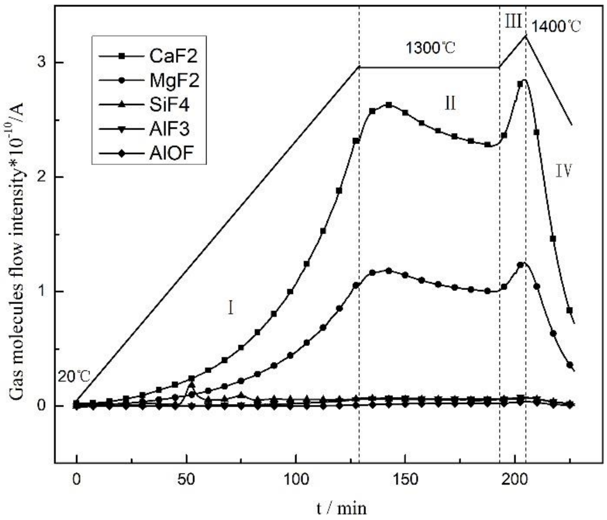

Figure S5 Mass spectrometry of fluorine-containing slag

Tables

Table S1 Main methods of determination of metallurgical slag performance

Properties	Unit	Physical symbol	Definition	Physical meaning	Measuring method
melting point	K	T	the temperature that solid translates into liquid completely	the melting temperature	hemisphere method
viscosity	Pa·S	Η	a measure of resistance to gradual deformation by shear stress or tensile stress	fluidity of melt	rotary cylinder method
surface tension	N·m	Σ	a contractive tendency of the surface of a liquid that allows it to resist an external force	ability to generate new interface	pull cylinder method
electrical conductivity	$S{\cdot}m^{-1}$	G	the reciprocal of electrical resistivity	a material's ability to conduct an electric current	electric bridge method
